# Portal Dissemination of *Fusarium graminearum* in a Patient with Acute Lymphoblastic Leukemia and Febrile Neutropenia

**DOI:** 10.3390/idr13010002

**Published:** 2021-01-01

**Authors:** Mary Gabriela Uscamayta, Alexandra Martin-Onraet, Karla Espinosa-Bautista, Roberto Herrera-Goepfert, Rigoberto Hernández-Castro, Carolina Perez-Jimenez

**Affiliations:** 1 Infectious Disease Department, National Institute of Cancer, Mexico City 14080, Mexico; gabriela-mary@hotmail.com (M.G.U.); alexitemaon@gmail.com (A.M.-O.); 2 Hematology Department, National Institute of Cancer, Mexico City 14080, Mexico; karlaadrianae@gmail.com; 3 Pathology Department, National Institute of Cancer, Mexico City 14080, Mexico; rhgoepfert@gmail.com; 4 Ecology of Pathogen Agents Department, General Hospital Manuel Gea González, Mexico City 14080, Mexico; rigo37@gmail.com

**Keywords:** *Fusarium graminearum*, portal dissemination, invasive fungal infection, rRNA sequence gene, febrile neutropenia

## Abstract

We present the case of a man with acute lymphoblastic leukemia and prolonged profound neutropenia, who developed an invasive infection by *Fusarium graminearum*, acquired via non-cutaneous entry, with gastrointestinal symptoms, sigmoid perforation and liver abscesses due to portal dissemination. The etiologic agent was identified using the 18S-ITS1-5.8S-ITS2-28S rRNA sequence gene, from a liver biopsy. The infection was resolved with surgical drainage and antifungal treatment based on voriconazole. As far as we know, there are no previous reports in the literature of cases of human infection due to *Fusarium graminearum*.

## 1. Introduction

In the last decades, there has been an increase in the incidence of invasive fungal infections (IFI), related to new immunosuppressive treatments and improved diagnosis with new molecular techniques and higher diagnostic yields [1,2]. We present the case of a male patient with acute lymphoblastic leukemia and prolonged profound neutropenia, who developed an invasive infection by *Fusarium graminearum*, acquired via non-cutaneous entry, presenting an atypical clinical course, gastrointestinal symptoms, sigmoid perforation and liver abscesses due to portal dissemination. The etiologic agent was identified using the 18S-ITS1-5.8S-ITS2-28S rRNA sequence gene from a liver biopsy. This is the first reported case of portal dissemination by *Fusarium* genus and the first report of *Fusarium graminearum* infection in humans.

## 2. Ethical Considerations

The case we submit for publication is about a single patient who provided his verbal consent for this purpose. We do not include any details or information that could lead to patient identification

## 3. Case Report

Male patient, 26 years old, kitchen assistant, originated from Taxco, Guerrero and living there until he emigrated to Texas, United States, in 2016, where he was diagnosed with B lymphoblastic leukemia in January 2017. He received four cycles of unspecified chemotherapy with apparent remission of the disease and came back to Mexico (Cuernavaca, Morelos). He was admitted to the National Institute of Cancer in Mexico City in November 2017, where he was diagnosed with early relapse. From November 2017 to January 2018, he received chemotherapy for relapse with BFM like protocol (L-Asparaginase, Vincristine, Daunorubicin, and Prednisone). He did not receive any antifungal prophylaxis. On day +23 he was presented with febrile neutropenia, with no etiologic agent identified. An empirical treatment of five days of Meropenem was administered, with resulting in fever remission. He was discharged 6 days after his admission, asymptomatic but with persistent neutropenia.

**Figure 1:**
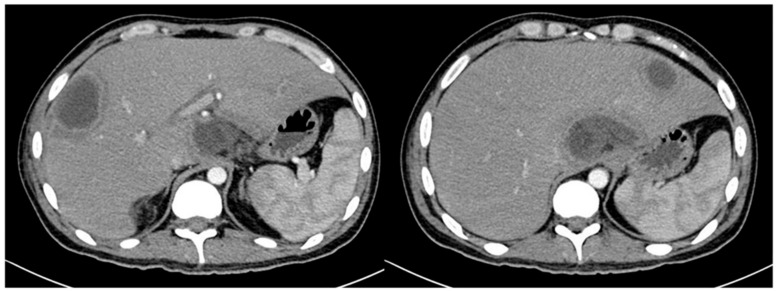
Abdominal CT scan with intravenous contrast, which shows: Diffuse and poorly defined hypodense lesions, one located in the liver segment II measuring 3.4 cm, another located in the caudate lobe measuring 7 cm, and another located in the hepatic segment VII with extension to segment VI measuring 7 cm; consistent with liver abscesses.

Two days later, he had come back with an acute abdomen, although afebrile. Vital constants at admission were: BP: 107/78 mmHg, HR: 90 bpm, RR: 20 rpm, T °: 36.5 °C, weight: 55 Kg, height: 1.62 m, BMI: 21 Kg/m2. His blood tests showed leukocytes of 0.32 × 10^3^/μL, absolute neutrophils 0.0032 x10^3^/μL, hemoglobin 5.7 g/dL, platelets 35 × 10^3^/μL, creatinine 0.66 mg/dL, ALT 174 IU/L, AST 82 IU/L, ALP 1004 IU/L, GGT 572 IU/L, total bilirubin 8 mg/dL, conjugated bilirubin 6.4 mg/dL, unconjugated bilirubin 1.62 mg/dL, LDH 287 IU/L, albumin 1.7 mg/dL, C reactive protein 8.4 mg/dL. The patient underwent exploratory laparotomy which revealed sigmoid perforation. Sigmoidectomy and colostomy were performed. The initial pathology report was diverticulum perforation. Forty-eight hours later, he presented moderate diffuse abdominal pain, and temperature > 38 °C. The blood cultures were negative. Administration of Meropenem, caspofungin and Filgastrim was empirically initiated. Contrast abdominal tomography (CT) showed diffuse liver lesions consistent with liver abscesses (Figure 1).

**Figure 2:**
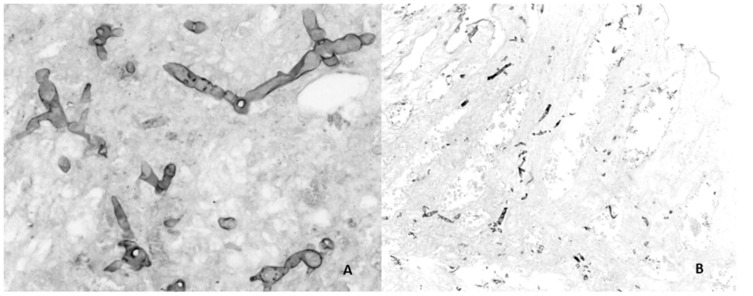
(**A**) Non-pigmented, separated hyphae with acute angle branching in liver biopsy tissue, Grocott methenamine silver-stained specimen, 40×. (**B**) Non-pigmented, separated hyphae with acute angle branching in intestinal tissue, Grocott methenamine silver-stained specimen, 10×.

A liver biopsy of the lesions was performed under ultrasound guidance; the aerobic, anaerobic and Sabouraud dextrose agar cultures were negative. The pathology showed granulomatous hepatitis with extensive necrosis; Grocott’s stain evidenced hyaline hyphae. The histopathology of the sigmoid surgical specimen was reviewed again and then reported as transmural ischemic necrosis, acute and chronic peritonitis, and positive Grocott’s stain with the same hyphae described in the liver (Figure 2).

Hyalohyphomycosis was concluded. The serum galactomannan was negative. Pulmonary involvement was not seen by tomography. The antifungal treatment was changed from caspofungin to intravenous Voriconazole on day 23. The patient had gradual clinical improvement the neutropenia was remitted and was discharged with a treatment of oral voriconazole 200 mg bid. He was, at that time, in complete remission of leukemia.

Three weeks later he was readmitted for abdominal pain in the right hypochondrium with fever without neutropenia; he reported bad adherence to oral antifungal treatment for economic reasons. The new abdominal CT showed the persistence of liver abscesses. Exploratory laparotomy plus drainage were performed finding abscesses in segments II, IV and VI. The anaerobic culture grew *Bacteroides fragilis*; he completed 14 days of Metronidazole. The fungal culture was negative. The pathology report was extensive granulomatous hepatitis with necrosis and negative Grocott’s stain. The patient received 2 weeks of intravenous Voriconazole plus 6 weeks of the same oral antifungal. At the end of treatment, the abdominal CT showed only one residual lesion of 16 mm. A timeline of the case is shown in Figure S1.

He did not present further complications of the infectious process. However, six months later, while undergoing hematopoietic stem-cell transplant evaluations, he presented a relapse of leukemia and he decided to stop the antineoplastic treatment. He died seven months later.

### Materials and Methods for Identification of the Causative Agent

**Figure 3:**
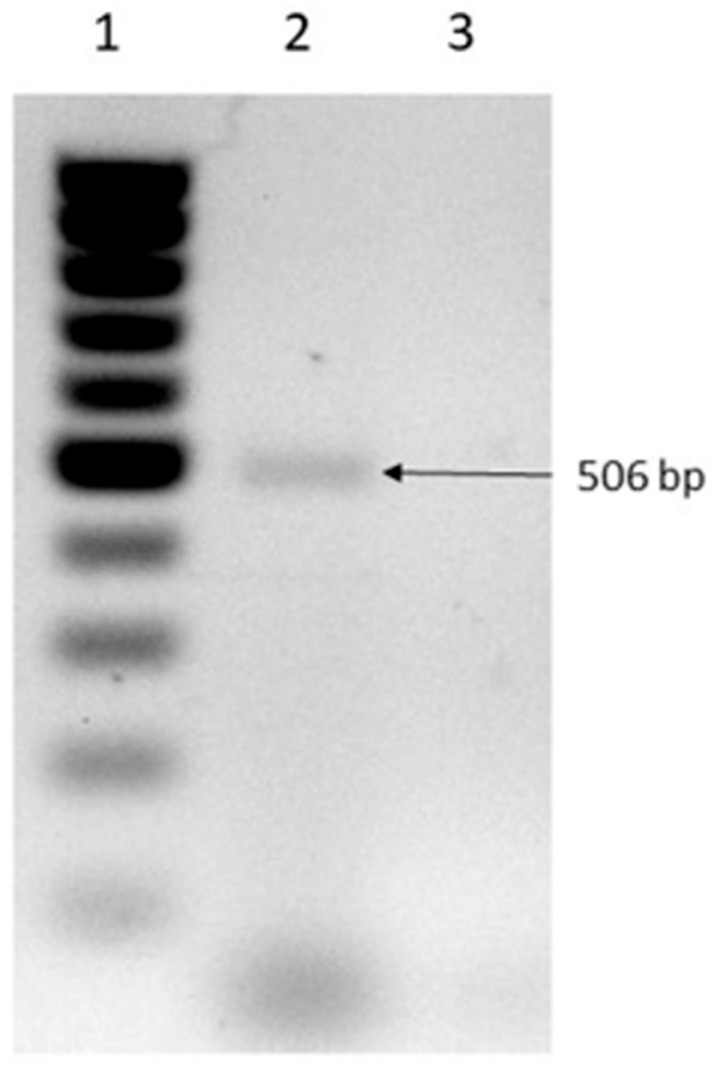
The 18S-ITS1-5.8S-ITS2-25S rRNA gene amplification. Lane 1: Molecular weight marker 100 bp DNA ladder; Lane 2: Liver biopsy sample; Lane 3: Negative control.

A paraffin block of the first liver biopsy was requested, and DNA extraction was performed. Genomic DNA was extracted from the paraffin-embedded tissue sample using a DNeasy blood and tissue kit (Qiagen, Ventura, CA, USA) according to the manufacturer’s instructions before preliminary removal of paraffin by extraction with xylene protocol. The molecular identification was performed by 18S-ITS1-5.8S-ITS2-25S rRNA gene amplification using a set of primers previously reported to identify fungi species (5′-TCCGTAGGTGAACCTTGCGG-3′) and ITS4 (5′-TCCTCCGCTTATTGATATGC-3′). A PCR product of 500 bp was amplified, purified and sequenced in both directions; nucleotide sequence was determined with Taq FS Dye Terminator Cycle Sequencing Fluorescence-Based Sequencing and analyzed on an Applied Biosystems 3730 DNA sequencing system (Foster City, CA, USA). The sequence was edited with Vector NTI program and homology search was performed in the GenBank database (nucleotide blast), finding a 100% identity with *Fusarium graminearum* complex strain HUT59 [3]. (Figure 3). The sequences used are shown in Supplementary Figure S2.

## 4. Discussion

In the last ten years, there has been an increasing incidence of disseminated filamentous fungal infections in patients with severe immunosuppression [1,2,4]. Hematological malignancies have a risk of 6.5% for IFI [5], mainly in the induction and consolidation phases. This increase is due to severe immunosuppression conditions by myelotoxic treatments and related clinical conditions but also to new technologies such as molecular biology [6,7] that have increased the diagnosis ability.

Hialohifomycetes are opportunistic filamentous fungi that usually cause infection after environmental exposure [6]. Infection occurs through inhalation or skin breakdown (wounds, burns) [8]. *Fusarium* spp. is the second cause of infections by filamentous fungi after *Aspergillus* spp. More than 50 *Fusarium* species have been identified, but only about 12 are associated with infection in humans. *F. incarnatum, F. moniliforme, F. oxysporum* and *F. solani* usually cause allergic reactions and superficial infections such as keratitis and onychomycosis in immunocompetent hosts. In immunocompromised patients, it can cause from locally invasive diseases such as sinusitis and pneumonia, to fungemia and disseminated disease [9,10]. In neutropenic patients, it usually manifests as fever not responding to empirical antibiotics. Profound and prolonged neutropenia is the most important risk factor for dissemination and death due to fusariosis [8,11]. The most frequently isolated species in this setting are *F. solani* (50%) and *F. oxysporum* (20%) [6,8].

*Fusarium graminearum* plays a major role as the etiological agent of one important disease of small grain cereals and corn. First identified in 1884 in England, it is considered a major threat to wheat and barley [12]. The fungi, transmitted by seed, can survive up to 10 years as spores or resting structures in and on the seed. In Mexico, *F. graminearum* has been found in Hidalgo, Jalisco, Mexico, Michoacán and Tlaxcala [13,14,15,16]. This fungus can produce several mycotoxins, which have shown abortive estrogenic, gastric (nausea and vomiting) and neurotoxic toxicity in swine, cattle, dogs, cats, and ducks [16,17]. Grain that has been infected with the fungus can be incorporated into basic diets and could be a source of infection in humans. So far, no infections due to *F. graminareum* have been reported. Considering the patient’s occupation (kitchen helper) and the high corn content in the Mexican diet, we supposed that the patient was colonized with *F. graminearum* through the daily ingestion and handling of corn. He possibly got the mould through oral ingestion, and symptoms were gastrointestinal due to portal dissemination, which explains why we did not document any fungemia or cutaneous infection, despite an invasive disease. Usually, in disseminated fusariosis, blood cultures are positive in more than 75% of cases [6,8].

In our patient, the diagnosis of hyalohyphomycosis was first made by pathologic specimen. The biopsy revealed the presence of septate hyaline hyphae 3–6 μm diameter, indistinguishable from other hyalohyphomycosis such as *Aspergillus, Acremonium* [*Cephalosporium*]*,* and *Pseudallescheria* [17]. Recently, the Mycotic Diseases Branch, from the Center for Disease Control and Prevention in Atlanta, described a new entity of invasive fungal infections (IFI), which they called “foodborne IFI”, in a recently published systematic review [18], foodborne IFI are infections acquired through ingestion of food or water contaminated with the fungi causing the infection. Reports describe molds and yeasts as etiologic agents. *Rhizopus* sp is the most frequently described mold, but one case of IFI due to *Fusarium moniliforme* is reported in a man with acute lymphoblastic leukemia who ingested cereals. The diagnosis of foodborne IFI is difficult, clinical presentation is usually atypical, and mainly gastrointestinal. Fungi can spoil food that is ingested, and it is suggested that this category of fungal infections could be related to food insecurity. The source of the fungi should be identified to be able to classify the IFI as foodborne, which is often difficult to the ubiquitous nature of these fungi. As previously mentioned, although we suspect that our patient acquired the infection orally and this could correspond to foodborne IFI, we cannot corroborate the above because we could not have samples of the food that the patient ingested prior to his hospitalization and we did not perform stool cultures.

Today, comparative identification strategies based on gene sequencing can be considered the new gold standard for the identification of fungal species. Gene sequencing is characterized by PCR amplification of the ITS region, and sequencing of the resulting amplicon (s). The ITS region can be amplified reliably for most fungi [19]. These molecular biology techniques provide better discrimination of species morphologically indistinguishable from *Fusarium* spp, and can be performed on different types of materials, including paraffin blocks, which is why it is considered an emerging and promising methodology to be used in the routine identification of *Fusarium* spp. In our case, no other fungal species were identified in the studied specimen or in any other specimen from the patient. The *Fusarium graminearum* complex includes 13 species; biogeographic data suggest that the majority of the species within the complex have evolutionary origins in the Southern hemisphere and Asia. According to the surveys to date, *F. austroamericanum*, *F. meridionale*, *F. cortaderiae*, and *F. brasilicum* appear to be endemic to South America; *F. acaciae-mearnsii* to Australia or less likely Africa; *F. asiaticum*, *F. vorosii* and *F. ussurianum* to Asia; *F. aethiopicum* to Africa; *F. boothii* and *F. mesoamericanum* to Central America; *F. gerlachii* to the US and *F. graminearum* sensu stricto (F. graminearum s.s.) it is the most cosmopolitan species and has been found in Asia, Africa, America, Europe, and Oceania [20].

Finally, the treatment in localized disease can benefit from surgical debridement especially in disseminated disease together with the use of systemic antifungals. *Fusarium* species are relatively resistant in vitro to most antifungal agents, showing different susceptibility patterns between species. In general, most *Fusarium* isolates have lower inhibitory concentrations (MIC) for Amphotericin B higher than *Aspergillus* species. *F. solani* and *F. verticillioides* are usually resistant to azoles and they have higher MIC for azoles than other *Fusarium* species. In contrast, *F. oxysporum* and *F. moniliforme* may be susceptible to voriconazole and posaconazole [9]. Echinocandins show the highest MIC values [21]. As far as we know there are no previous reports in the medical literature of identification of *F.graminearum* as a pathogen in humans; therefore antifungal therapy was initiated based on is the antifungals reported in the literature for the Fusarium species and on the clinical response.

## 5. Conclusions

Prolonged profound neutropenia and alteration of the intestinal flora, secondary to myelotoxic chemotherapies and environmental exposure in patients with hematological neoplasms, are important risk factors for the development of invasive infections by filamentous fungi. This is the first reported case of invasive fungal infection by *F. graminearum* and the first case of portal dissemination of *Fusarium* spp. The anamnesis and exposure to environmental factors are important during clinical evaluation. *F.graminearum* was not identifiable by traditional methods such as culture and pathology. Access to molecular biology is extremely valuable to increase the performance of diagnostic tests in the identification of new species such as the *F. graminearum* reported in our paper.

## Data Availability

Data is contained within the article or supplementary material.

## Supplementary Materials

The following are available online at https://www.mdpi.com/2036-7449/13/1/2/s1, Supplementary material 1 (Figure S1) is a timeline of the present case. Supplementary material 2 (Figure S2) is a figure with the genetic sequences used for the identification of the etiologic agent (*Fusarium graminearum* complex).
