# Supplementary material for: Portal Dissemination of Fusarium graminearum in a Patient with Acute Lymphoblastic Leukemia and Febrile Neutropenia
Source: Infect Dis Rep. 2021 Jan 1;13(1):11–7. doi: 10.3390/idr13010002 (PMC7838889; doi:10.3390/idr13010002)
Supplement: Supplementary file 1 [file idr-13-00002-s001.zip › idr-1000003-supplementary/Figure S1 Timeline F. graminearum.pdf]

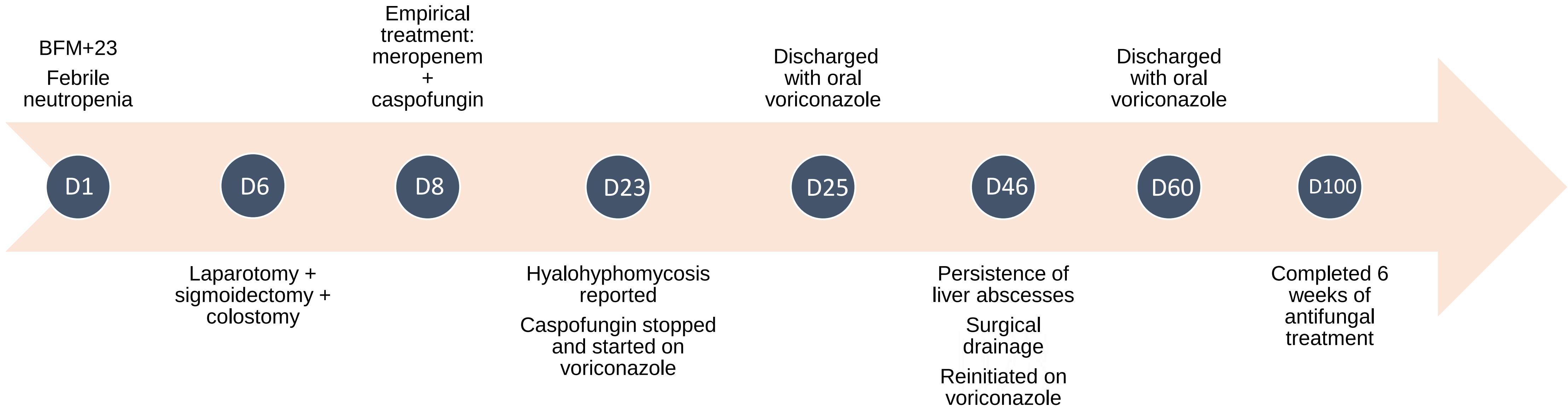

Figure S1: Timeline of patient's treatment from day 1 (D1) to day 100 (D100).
BFM: BFM like protocol (L-asparaginase, vincristine, daunorubicin and prednisone
