# Supplementary material for: Portal Dissemination of Fusarium graminearum in a Patient with Acute Lymphoblastic Leukemia and Febrile Neutropenia
Source: Infect Dis Rep. 2021 Jan 1;13(1):11–7. doi: 10.3390/idr13010002 (PMC7838889; doi:10.3390/idr13010002)
Supplement: Supplementary file 1 [file idr-13-00002-s001.zip › idr-1000003-supplementary/Figure S2_F. graminearum complex sequences.docx]

1 100

F. cortaderiae EFA 6FCMG (1) CTTGGTCATTTAGAGGAAGTAAAAG-TCGTAACAAGGTCTCCGTTGGTGAACCAGCGGAGGGATCATTACCGAGTTTACAACTCCCAAACCCCTGTGAAC

F meridionale NRRL28436 (1) --------------------------TCGTAACAAGGTCTCCGTTGGTGAACCAGCGGAGGGATCATTACCGAGTTTACAACTCCCAAACCCCTGTGAAC

F. brasilicum NRRL 31238 (1) --------------------------TCGTAACAAGGTCTCCGTTGGTGAACCAGCGGAGGGATCATTACCGAGTTTACAACTCCCAAACCCCTGTGAAC

F. mesoamericanum NRRL29148 (1) --------------------------TCGTAACAAGGTCTCCGTTGGTGAACCAGCGGAGGGATCATTACCGAGTTTACAACTCCCAAACCCCTGTGAAC

F. acaciae-mearnsii NRRL26752 (1) --------------------------TCGTAACAAGGTCTCCGTTGGTGAACCAGCGGAGGGATCATTACCGAGTTTACAACTCCCAAACCCCTGTGAAC

F. aethiopicum NRRL 46722 (1) --------------------------TCGTAACAAGGTCTCCGTTGGTGAACCAGCGGAGGGATCATTACCGAGTTTACAACTCCCAAACCCCTGTGAAC

F. boothii PM3 (1) -----------TTTGGAAGTAAAAG-TCGTAACAAGGTCTCCGTTGGTGAACCAGCGGAGGGATCATTACCGAGTTTACAACTCCCAAACCCCTGTGAAC

F. austroamericanum JCP40 (1) -------------------------------------------TTGGTGAACCAGCGGAGGGATCATTACCGAGTTTACAACTCCCAAACCCCTGTGAAC

F. cerealis 11080 (1) -----------------------AAGTCGTAACAAGGTCTCCGTTGGTGAACCAGCGGAGGGATCATTACCGAGTTTACAACTCCCAAACCCCTGTGAAC

F. asiaticum ROB2 (1) -----------------------------------------------------TGCGGAGGGATCATTACCGAGTTTACAACTCCCAAACCCCTGTGAAC

F. cerealis AT3-6 (1) ---------------------------------------------------------GAGGGATCATTACCGAGTTTACAACTCCCAAACCCCTGTGAAC

F. vorosii TMSX103 (1) -------------TGGAAGTAAAAG-TCGTAACAAGGTCTCCGTTGGTGAACCAGCGGAGGGATCATTACCGAGTTTACAACTCCCAAACCCCTGTGAAC

F. pseudograminearum ZF28 (1) ----------------------------------------------------------------CATTACCGAGTTTACAACTCCCAAACCCCTGTGAAC

F. culmorum WF3 (1) ------------------GTAAAAG-TCGTAACAAGGTCTCCGTTGGTGAACCAGCGGAGGGATCATTACCGAGTTTACAACTCCCAAACCCCTGTGAAC

F. ussurianum NRRL 45681 (1) --------------------------TCGTAACAAGGTCTCCGTTGGTGAACCAGCGGAGGGATCATTACCGAGTTTACAACTCCCAAACCCCTGTGAAC

F. graminearum HUT59 (1) ---------------------AAAA-TCGTAACAAGGTCTCCGTTGGTGAACCAGCGGAGGGATCATTACCGAGTTTACAACTCCCAAACCCCTGTGAAC

F. graminearum W430 (1) ---------GCCTGGGATGTAAAAAATCGTAACAAGGTCTCCGTTGGTGAACCAGCGGAGGGATCATTACCGAGTTTACAACTCCCAAACCCCTGTGAAC

InCan (1) ---------------------AAAA-TCGTAACAAGGTCTCCGTTGGTGAACCAGCGGAGGGATCATTACCGAGTTTACAACTCCCAAACCCCTGTGAAC

Consensus (1) TCGTAACAAGGTCTCCGTTGGTGAACCAGCGGAGGGATCATTACCGAGTTTACAACTCCCAAACCCCTGTGAAC

101 200

F. cortaderiae EFA 6FCMG (100) ATACCTTATGTTGCCTCGGCGGATCAGCCCGCGCCCCGTAAAAAGGGACGGCCCGCCGCAGGAACCCTAAACTCTGTTTTTAGTGGAACTTCTGAGTATA

F meridionale NRRL28436 (75) ATACCTTATGTTGCCTCGGCGGATCAGCCCGCGCCCCGTAAAAAGGGACGGCCCGCCGCAGGAACCCTAAACTCTGTTTTTAGTGGAACTTCTGAGTATA

F. brasilicum NRRL 31238 (75) ATACCTTATGTTGCCTCGGCGGATCAGCCCGCGCCCCGTAAAAAGGGACGGCCCGCCGCAGGAACCCTAAACTCTGTTTTTAGTGGAACTTCTGAGTATA

F. mesoamericanum NRRL29148 (75) ATACCTTATGTTGCCTCGGCGGATCAGCCCGCGCCCCGTAAAAAGGGACGGCCCGCCGCAGGAACCCTAAACTCTGTTTTTAGTGGAACTTCTGAGTATA

F. acaciae-mearnsii NRRL26752 (75) ATACCTTATGTTGCCTCGGCGGATCAGCCCGCGCCCCGTAAAAAGGGACGGCCCGCCGCAGGAACCCTAAACTCTGTTTTTAGTGGAACTTCTGAGTATA

F. aethiopicum NRRL 46722 (75) ATACCTTATGTTGCCTCGGCGGATCAGCCCGCGCCCCGTAAAAAGGGACGGCCCGCCGCAGGAACCCTAAACTCTGTTTTTAGTGGAACTTCTGAGTATA

F. boothii PM3 (89) ATACCTTATGTTGCCTCGGCGGATCAGCCCGCGCCCCGTAAAAAGGGACGGCCCGCCGCAGGAACCCTAAACTCTGTTTTTAGTGGAACTTCTGAGTATA

F. austroamericanum JCP40 (58) ATACCTTATGTTGCCTCGGCGGATCAGCCCGCGCCCCGTAAAAAGGGACGGCCCGCCGCAGGAACCCTAAACTCTGTTTTTAGTGGAACTTCTGAGTATA

F. cerealis 11080 (78) ATACCTTATGTTGCCTCGGCGGATCAGCCCGCGCCCCGTAAAAAGGGACGGCCCGCCGCAGGAACCCTAAACTCTGTTTTTAGTGGAACTTCTGAGTATA

F. asiaticum ROB2 (48) ATACCTTATGTTGCCTCGGCGGATCAGCCCGCGCCCCGTAAAAAGGGACGGCCCGCCGCAGGAACCCTAAACTCTGTTTTTAGTGGAACTTCTGAGTATA

F. cerealis AT3-6 (44) ATACCTTATGTTGCCTCGGCGGATCAGCCCGCGCCCCGTAAAAAGGGACGGCCCGCCGCAGGAACCCTAAACTCTGTTTTTAGTGGAACTTCTGAGTATA

F. vorosii TMSX103 (87) ATACCTTATGTTGCCTCGGCGGATCAGCCCGCGCCCCGTAAAAAGGGACGGCCCGCCGCAGGAACCCTAAACTCTGTTTTTAGTGGAACTTCTGAGTATA

F. pseudograminearum ZF28 (37) ATACCTTATGTTGCCTCGGCGGATCAGCCCGCGCCCCGTAAAAAGGGACGGCCCGCCGCAGGAACCCCAAACTCTGTTTTTAGTGGAACTTCTGAGTATA

F. culmorum WF3 (82) ATACCTTATGTTGCCTCGGCGGATCAGCCCGCGCCCCGTAAAAAGGGACGGCCCGCCGCAGGAACCCTAAACTCTGTTTTTAGTGGAACTTCTGAGTATA

F. ussurianum NRRL 45681 (75) ATACCTTATGTTGCCTCGGCGGATCAGCCCGCGCCCCGTAAAAAGGGACGGCCCGCCGCAGGAACCCTAAACTCTGTTTTTAGTGGAACTTCTGAGTATA

F. graminearum HUT59 (79) ATACCTTATGTTGCCTCGGCGGATCAGCCCGCGCCCCGTAAAAAGGGACGGCCCGCCGCAGGAACCCTAAACTCTGTTTTTAGTGGAACTTCTGAGTATA

F. graminearum W430 (92) ATACCTTATGTTGCCTCGGCGGATCAGCCCGCGCCCCGTAAAAAGGGACGGCCCGCCGCAGGAACCCTAAACTCTGTTTTTAGTGGAACTTCTGAGTATA

InCan (79) ATACCTTATGTTGCCTCGGCGGATCAGCCCGCGCCCCGTAAAAAGGGACGGCCCGCCGCAGGAACCCTAAACTCTGTTTTTAGTGGAACTTCTGAGTATA

Consensus (101) ATACCTTATGTTGCCTCGGCGGATCAGCCCGCGCCCCGTAAAAAGGGACGGCCCGCCGCAGGAACCCTAAACTCTGTTTTTAGTGGAACTTCTGAGTATA

201 300

F. cortaderiae EFA 6FCMG (200) AAAAACAAATAAATCAAAACTTTCAACAACGGATCTCTTGGTTCTGGCATCGATGAAGAACGCAGCAAAATGCGATAAGTAATGTGAATTGCAGAATTCA

F meridionale NRRL28436 (175) AAAAACAAATAAATCAAAACTTTCAACAACGGATCTCTTGGTTCTGGCATCGATGAAGAACGCAGCAAAATGCGATAAGTAATGTGAATTGCAGAATTCA

F. brasilicum NRRL 31238 (175) AAAAACAAATAAATCAAAACTTTCAACAACGGATCTCTTGGTTCTGGCATCGATGAAGAACGCAGCAAAATGCGATAAGTAATGTGAATTGCAGAATTCA

F. mesoamericanum NRRL29148 (175) AAAAACAAATAAATCAAAACTTTCAACAACGGATCTCTTGGTTCTGGCATCGATGAAGAACGCAGCAAAATGCGATAAGTAATGTGAATTGCAGAATTCA

F. acaciae-mearnsii NRRL26752 (175) AAAAACAAATAAATCAAAACTTTCAACAACGGATCTCTTGGTTCTGGCATCGATGAAGAACGCAGCAAAATGCGATAAGTAATGTGAATTGCAGAATTCA

F. aethiopicum NRRL 46722 (175) AAAAACAAATAAATCAAAACTTTCAACAACGGATCTCTTGGTTCTGGCATCGATGAAGAACGCAGCAAAATGCGATAAGTAATGTGAATTGCAGAATTCA

F. boothii PM3 (189) AAAAACAAATAAATCAAAACTTTCAACAACGGATCTCTTGGTTCTGGCATCGATGAAGAACGCAGCAAAATGCGATAAGTAATGTGAATTGCAGAATTCA

F. austroamericanum JCP40 (158) AAAAACAAATAAATCAAAACTTTCAACAACGGATCTCTTGGTACTGGCATCGATGAAGAACGCAGCAAA-TGCGATAAGTAATGTGAATTGCAGAATTCA

F. cerealis 11080 (178) AAAAACAAATAAATCAAAACTTTCAACAACGGATCTCTTGGTTCTGGCATCGATGAAGAACGCAGCAAAATGCGATAAGTAATGTGAATTGCAGAATTCA

F. asiaticum ROB2 (148) AAAAACAAATAAATCAAAACTTTCAACAACGGATCTCTTGGTTCTGGCATCGATGAAGAACGCAGCAAAATGCGATAAGTAATGTGAATTGCAGAATTCA

F. cerealis AT3-6 (144) AAAAACAAATAAATCAAAACTTTCAACAACGGATCTCTTGGTTCTGGCATCGATGAAGAACGCAGCAAAATGCGATAAGTAATGTGAATTGCAGAATTCA

F. vorosii TMSX103 (187) AAAAACAAATAAATCAAAACTTTCAACAACGGATCTCTTGGTTCTGGCATCGATGAAGAACGCAGCAAAATGCGATAAGTAATGTGAATTGCTGAATTCA

F. pseudograminearum ZF28 (137) AAAAACAAATAAATCAAAACTTTCAACAACGGATCTCTTGGTTCTGGCATCGATGAAGAACGCAGCAAAATGCGATAAGTAATGTGAATTGCAGAATTCA

F. culmorum WF3 (182) AAAAACAAATAAATCAAAACTTTCAACAACGGATCTCTTGGTTCTGGCATCGATGAAGAACGCAGCAAAATGCGATAAGTAATGTGAATTGCAGAATTCA

F. ussurianum NRRL 45681 (175) AAAAACAAATAAATCAAAACTTTCAACAACGGATCTCTTGGTTCTGGCATCGATGAAGAACGCAGCAAAATGCGATAAGTAATGTGAATTGCAGAATTCA

F. graminearum HUT59 (179) AAAAACAAATAAATCAAAACTTTCAACAACGGATCTCTTGGTTCTGGCATCGATGAAGAACGCAGCAAAATGCGATAAGTAATGTGAATTGCAGAATTCA

F. graminearum W430 (192) AAAAACAAATAAATCAAAACTTTCAACAACGGATCTCTTGGTTCTGGCATCGATGAAGAACGCAGCAAAATGCGATAAGTAATGTGAATTGCAGAATTCA

InCan (179) AAAAACAAATAAATCAAAACTTTCAACAACGGATCTCTTGGTTCTGGCATCGATGAAGAACGCAGCAAAATGCGATAAGTAATGTGAATTGCAGAATTCA

Consensus (201) AAAAACAAATAAATCAAAACTTTCAACAACGGATCTCTTGGTTCTGGCATCGATGAAGAACGCAGCAAAATGCGATAAGTAATGTGAATTGCAGAATTCA

301 400

F. cortaderiae EFA 6FCMG (300) GTGAATCATCGAATCTTTGAACGCACATTGCGCCCGCCAGTATTCTGGCG GGCATGCCTGTTCGAGCGTCATTTCAACCCTCAAGCCCAGCTTGGTGTTG

F meridionale NRRL28436 (275) GTGAATCATCGAATCTTTGAACGCACATTGCGCCCGCCAGTATTCTGGCG GGCATGCCTGTTCGAGCGTCATTTCAACCCTCAAGCCCAGCTTGGTGTTG

F. brasilicum NRRL 31238 (275) GTGAATCATCGAATCTTTGAACGCACATTGCGCCCGCCAGTATTCTGGCG GGCATGCCTGTTCGAGCGTCATTTCAACCCTCAAGCCCAGCTTGGTGTTG

F. mesoamericanum NRRL29148 (275) GTGAATCATCGAATCTTTGAACGCACATTGCGCCCGCCAGTATTCTGGCG GGCATGCCTGTTCGAGCGTCATTTCAACCCTCAAGCCCAGCTTGGTGTTG

F. acaciae-mearnsii NRRL26752 (275) GTGAATCATCGAATCTTTGAACGCACATTGCGCCCGCCAGTATTCTGGCG GGCATGCCTGTTCGAGCGTCATTTCAACCCTCAAGCCCAGCTTGGTGTTG

F. aethiopicum NRRL 46722 (275) GTGAATCATCGAATCTTTGAACGCACATTGCGCCCGCCAGTATTCTGGCG GGCATGCCTGTTCGAGCGTCATTTCAACCCTCAAGCCCAGCTTGGTGTTG

F. boothii PM3 (289) GTGAATCATCGAATCTTTGAACGCACATTGCGCCCGCCAGTATTCTGGCG GGCATGCCTGTTCGAGCGTCATTTCAACCCTCAAGCCCAGCTTGGTGTTG

F. austroamericanum JCP40 (257) GTGAATCATCGAATCTTTGACAGCACATTGCGCCCGCCAGTATTCTGGCG GGCATGCCTGTTCGAGCGTCATTTCAACCCTCAAGCCCAGCTTGGTGTTG

F. cerealis 11080 (278) GTGAATCATCGAATCTTTGAACGCACATTGCGCCCGCCAGTATTCTGGCG GGCATGCCTGTTCGAGCGTCATTTCAACCCTCAAGCCCAGCTTGGTGTTG

F. asiaticum ROB2 (248) GTGAATCATCGAATCTTTGAACGCACATTGCGCCCGCCAGTATTCTGGCG GGCATGCCTGTTCGAGCGTCATTTCAACCCTCAAGCCCAGCTTGGTGTTG

F. cerealis AT3-6 (244) GTGAATCATCGAATCTTTGAACGCACATTGCGCCCGCCAGTATTCTGGCG GGCATGCCTGTTCGAGCGTCATTTCAACCCTCAAGCCCAGCTTGGTGTTG

F. vorosii TMSX103 (287) GTGAATCATCGAATCTTTGAACGCACATTGCGCCCGCCAGTATTCTGGCG GGCATGCCTGTTCGAGCGTCATTTCAACCCTCAAGCCCAGCTTGGTGTTG

F. pseudograminearum ZF28 (237) GTGAATCATCGAATCTTTGAACGCACATTGCGCCCGCCAGTATTCTGGCG GGCATGCCTGTTCGAGCGTCATTTCAACCCTCAAGCCCAGCTTGGTGTTG

F. culmorum WF3 (282) GTGAATCATCGAATCTTTGAACGCACATTGCGCCCGCCAGTATTCTGGCG GGCATGCCTGTTCGAGCGTCATTTCAACCCTCAAGCCCAGCTTGGTGTTG

F. ussurianum NRRL 45681 (275) GTGAATCATCGAATCTTTGAACGCACATTGCGCCCGCCAGTATTCTGGCG GGCATGCCTGTTCGAGCGTCATTTCAACCCTCAAGCCCAGCTTGGTGTTG

F. graminearum HUT59 (279) GTGAATCATCGAATCTTTGAACGCACATTGCGCCCGCCAGTATTCTGGCG GGCATGCCTGTTCGAGCGTCATTTCAACCCTCAAGCCCAGCTTGGTGTTG

F. graminearum W430 (292) GTGAATCATCGAATCTTTGAACGCACATTGCGCCCGCCAGTATTCTGGCG GGCATGCCTGTTCGAGCGTCATTTCAACCCTCAAGCCCAGCTTGGTGTTG

InCan (279) GTGAATCATCGAATCTTTGAACGCACATTGCGCCCGCCAGTATTCTGGCG GGCATGCCTGTTCGAGCGTCATTTCAACCCTCAAGCCCAGCTTGGTGTTG

Consensus (301) GTGAATCATCGAATCTTTGAACGCACATTGCGCCCGCCAGTATTCTGGCG GGCATGCCTGTTCGAGCGTCATTTCAACCCTCAAGCCCAGCTTGGTGTTG

401 500

F. cortaderiae EFA 6FCMG (400) GGAGCTGCAGTCCTGCTGCACTCCCCAAATACATTGGCGGTCACGTCGAGCTTCCATAGCG-TAGTAATTTACACATCGTTACTGGTAATCGTCGCGGCC

F meridionale NRRL28436 (375) GGAGCTGCAGTCCTGCTGCACTCCCCAAATACATTGGCGGTCACGTCGAGCTTCCATAGCG-TAGTAATTTACACATCGTTACTGGTAATCGTCGCGGCC

F. brasilicum NRRL 31238 (375) GGAGCTGCAGTCCTGCTGCACTCCCCAAATACATTGGCGGTCACGTCGAGCTTCCATAGCG-TAGTAATTTACACATCGTTACTGGTAATCGTCGCGGCC

F. mesoamericanum NRRL29148 (375) GGAGCTGCAGTCCTGCTGCACTCCCCAAATACATTGGCGGTCACGTCGAGCTTCCATAGCG-TAGTAATTTACACATCGTTACTGGTAATCGTCGCGGCC

F. acaciae-mearnsii NRRL26752 (375) GGAGCTGCAGTCCTGCTGCACTCCCCAAATACATTGGCGGTCACGTCGAGCTTCCATAGCG-TAGTAATTTACACATCGTTACTGGTAATCGTCGCGGCC

F. aethiopicum NRRL 46722 (375) GGAGCTGCAGTCCTGCTGCACTCCCCAAATACATTGGCGGTCACGTCGAGCTTCCATAGCG-TAGTAATTTACACATCGTTACTGGTAATCGTCGCGGCC

F. boothii PM3 (389) GGAGCTGCAGTCCTGCTGCACTCCCCAAATACATTGGCGGTCACGTCGAGCTTCCATAGCG-TAGTAATTTACACATCGTTACTGGTAATCGTCGCGGCC

F. austroamericanum JCP40 (357) GGAGCTGCAGTCCTGCTGCACTCCCCAAATACATTGGCGGTCACGTCGAGCTTCCATAGCGGTAGTAATTTACACATCGTTACTGGTAATCGACGCGGCC

F. cerealis 11080 (378) GGAGCTGCAGTCCTGCTGCACTCCCCAAATACATTGGCGGTCACGTCGAGCTTCCATAGCG-TAGTAATTTACACATCGTTACTGGTAATCGTCGCGGCC

F. asiaticum ROB2 (348) GGAGCTGCAGTCCTGCTGCACTCCCCAAATACATTGGCGGTCACGTCGAGCTTCCATAGCG-TAGTAATTTACACATCGTTACTGGTAATCGTCGCGGCC

F. cerealis AT3-6 (344) GGAGCTGCAGTCCTGCTGCACTCCCCAAATACATTGGCGGTCACGTCGAGCTTCCATAGCG-TAGTAATTTACATATCGTTACTGGTAATCGTCGCGGCC

F. vorosii TMSX103 (387) GGAGCTGCAGTCCTGCTGCACTCCCCAAATACATTGGCGGTCACGTCGAGCTTCCATAGCG-TAGTAATTTACATATCGTTACTGGTAATCGTCGCGGCC

F. pseudograminearum ZF28 (337) GGAGCTGC-GTCC-GC-GCACTCCCCAAATACATTGGCGGTCACGTCGAGCTTCCATAGCG-TAGTAATTTACATATCGTTACTGGTAATCGTCGCGGCC

F. culmorum WF3 (382) GGAGCTGCAGTCCTGCTGCACTCCCCAAATACATTGGCGGTCACGTCGAGCTTCCATAGCG-TAGTAATTTACACATCGTTACTGGTAATCGTCGCGGCC

F. ussurianum NRRL 45681 (375) GGAGCTGCAGTCCTGCTGCACTCCCCAAATACATTGGCGGTCACGTCGAGCTTCCATAGCG-TAGTAATTTACACATCGTTACTGGTAATCGTCGCGGCC

F. graminearum HUT59 (379) GGAGCTGCAGTCCTGCTGCACTCCCCAAATACATTGGCGGTCACGTCGAGCTTCCATAGCG-TAGTAATTTACACATCGTTACTGGTAATCGTCGCGGCC

F. graminearum W430 (392) GGAGCTGCAGTCCTGCTGCACTCCCCAAATACATTGGCGGTCACGTCGAGCTTCCATAGCG-TAGTAATTTACACATCGTTACTGGTAATCGTCGCGGCC

InCan (379) GGAGCTGCAGTCCTGCTGCACTCCCCAAATACATTGGCGGTCACGTCGAGCTTCCATAGCG-TAGTAATTTACACATCGTTACTGGTAATCGTCGCGGCC

Consensus (401) GGAGCTGCAGTCCTGCTGCACTCCCCAAATACATTGGCGGTCACGTCGAGCTTCCATAGCG TAGTAATTTACACATCGTTACTGGTAATCGTCGCGGCC

501 600

F. cortaderiae EFA 6FCMG (499) ACGCCGTTAAACCCCAACTTCTGAATGTTGACCTCGGATCAGGTAGGAATACCCGCTGAACTTAAGCATATCAA--------------------------

F meridionale NRRL28436 (474) ACGCCGTTAAACCCCAACTTCTGAATGTTGACCTCGGATCAGGTAGGAATACCCGCTGAACTTAAGCATATCAATAAGCGGAGGAAAAGAAACCAACAGG

F. brasilicum NRRL 31238 (474) ACGCCGTTAAACCCCAACTTCTGAATGTTGACCTCGGATCAGGTAGGAATACCCGCTGAACTTAAGCATATCAATAAGCGGAGGAAAAGAAACCAACAGG

F. mesoamericanum NRRL29148 (474) ACGCCGTTAAACCCCAACTTCTGAATGTTGACCTCGGATCAGGTAGGAATACCCGCTGAACTTAAGCATATCAATAAGCGGAGGAAAAGAAACCAACAGG

F. acaciae-mearnsii NRRL26752 (474) ACGCCGTTAAACCCCAACTTCTGAATGTTGACCTCGGATCAGGTAGGAATACCCGCTGAACTTAAGCATATCAATAAGCGGAGGAAAAGAAACCAACAGG

F. aethiopicum NRRL 46722 (474) ACGCCGTTAAACCCCAACTTCTGAATGTTGACCTCGGATCAGGTAGGAATACCCGCTGAACTTAAGCATATCAATAAGCGGAGGAAAAGAAACCAACAGG

F. boothii PM3 (488) ACGCCGTTAAACCCCAACTTCTGAATGTTGACCTCGGATCAGGTAGGAATACCCGCTGAACTTAAGCATATCAATAAGCGGAGGAAAA------------

F. austroamericanum JCP40 (457) ACGCCGTTAAACCCCAACTTCTGAATGTTGACCTCGGATCA---AGGAATACCCGCTGAACTTAAGCATATCAATAAG----------------------

F. cerealis 11080 (477) ACGCCGTTAAACCCCAACTTCTGAATGTTGACCTCGGATCAGGTAGGAATACCCGCTGAACTTAAGCATATCAATAAGCGGAGGAAAAGAAACCAACAGG

F. asiaticum ROB2 (447) ACGCCGTTAAACCCCAACTTCTGAATGTTGACCTCGGATCAGGTAGGAATACCCGCTGAACTTAAGCATATCAATA------------------------

F. cerealis AT3-6 (443) ACGCCGTTAAACCCCAACTTCTGAATGTTGACCTCGGATCAGGTAGGAATACCCGCTGAACTTAAGCATATCAATAAGCGGAGGAA--------------

F. vorosii TMSX103 (486) ACGCCGTTAAACCCCAACTTCTGAATGTTGACCTCGGCTCAGGTAGG-----------------------------------------------------

F. pseudograminearum ZF28 (433) ACGCCGTTAAACCCCAACTTCTGAATGTTGACCTCGGATCAGGTAGGAATACCCGCTGAACTTAAGC---------------------------------

F. culmorum WF3 (481) ACGCCGTTAAACCCCAACTTCTGAATGTTGACCTCGGATCAGGTAGGAATACCCGCTGAACTT-------------------------------------

F. ussurianum NRRL 45681 (474) ACGCCGTTAAACCCCAACTTCTGAATGTTGACCTCGGATCAGGTAGGAATACCCGCTGAACTTAAGCATATCAATAAGCGGAGGAAAAGAAACCAACAGG

F. graminearum HUT59 (478) ACGCCGTTAAACCCCAACTTCTGAATGT------------------------------------------------------------------------

F. graminearum W430 (491) ACGCCGTTAAACCCCAACTTCTGAATGTTGACCTCGGATCAGGTAGGAATACCCGCTGAACTTAAGCATATCATAGGCGC--------------------

InCan (478) ACGCCGTTAAACCCCAACTTCTGAATGT------------------------------------------------------------------------

Consensus (501) ACGCCGTTAAACCCCAACTTCTGAATGTTGACCTCGGATCAGGTAGGAATACCCGCTGAACTTAAGCATATCAATAAGCGGAGGAA

**Figure S2**. Genetic sequences used for the identification of the etiologic agent (*Fusarium graminearum* complex.

Multiples aliment was established using the ClustalW algorithms mediante el programa Vector NTI advanced.
